# Sindbis Virus Vaccine Platform: A Promising Oncolytic Virus-Mediated Approach for Ovarian Cancer Treatment

**DOI:** 10.3390/ijms25052925

**Published:** 2024-03-02

**Authors:** Christine Pampeno, Silvana Opp, Alicia Hurtado, Daniel Meruelo

**Affiliations:** 1 Department of Pathology, NYU Grossman School of Medicine, New York University, New York, NY 10016, USA; 2 BioNTech, Cambridge, MA 02139, USA

**Keywords:** Sindbis virus, oncolytic virus, ovarian cancer, immunotherapy

## Abstract

This review article provides a comprehensive overview of a novel Sindbis virus vaccine platform as potential immunotherapy for ovarian cancer patients. Ovarian cancer is the most lethal of all gynecological malignancies. The majority of high-grade serous ovarian cancer (HGSOC) patients are diagnosed with advanced disease. Current treatment options are very aggressive and limited, resulting in tumor recurrences and 50–60% patient mortality within 5 years. The unique properties of armed oncolytic Sindbis virus vectors (SV) in vivo have garnered significant interest in recent years to potently target and treat ovarian cancer. We discuss the molecular biology of Sindbis virus, its mechanisms of action against ovarian cancer cells, preclinical in vivo studies, and future perspectives. The potential of Sindbis virus-based therapies for ovarian cancer treatment holds great promise and warrants further investigation. Investigations using other oncolytic viruses in preclinical studies and clinical trials are also presented.

## 1. Introduction

### 1.1. Ovarian Cancer as a Significant Health Concern

Ovarian cancer is one of the most common and lethal types of gynecological cancers with an overall survival rate of 50 percent that has not changed significantly for several decades [1,2,3]. Although it can occur at any age, it primarily affects women who have gone through menopause. The estimated rate of new cases and death in 2023 are 19,710 and 13,720, respectively [4]. Due to the absence of noticeable symptoms, ovarian cancer often goes undetected in its early stages and hence is referred to as the “silent killer” [5,6,7,8]. As the disease progresses, symptoms include abdominal bloating or swelling, difficulty eating or feeling full quickly, pelvic pain and fatigue. These symptoms can be indicative of other conditions as well, which makes early detection and accurate diagnosis challenging. As a result, patients are frequently diagnosed at an advanced stage when tumors have already spread beyond the ovaries. Thus, metastasis presents the greatest therapeutic challenge, restricting successful treatment of patients and dramatically reducing the overall survival rate [9,10].

There are different types of ovarian cancer, with epithelial ovarian cancer being the most common form, accounting for about 90% of cases [9,11,12,13,14,15]. Highly aggressive high-grade serous ovarian carcinoma (HGSOC) is the most common epithelial subtype with a tendency to develop early chemotherapy resistance. HGSOC presents with various molecular abnormalities, especially TP53 mutations observed in the majority of tumors [16]. Other less common types include germ cell tumors and stromal tumors, which develop in the cells that produce eggs and hormones within the ovary. The exact cause of ovarian cancer is not well understood, but certain factors have been identified that may increase a woman’s risk of developing the disease. These risk factors include a family history of ovarian or breast cancer, certain inherited gene mutations (such as TP53, BRCA1, and BRCA2), increasing age, obesity, and certain hormonal factors [12,13,17,18].

### 1.2. Current Treatment Challenges and Limitations

Current treatment strategies typically involve a combination of surgery, systemic chemotherapy, and sometimes radiation therapy. Surgery, which is often the first line of treatment, aims to remove as much tumor tissue as possible (referred to as “debulking” [19,20]), which includes removal of one or both ovaries, fallopian tubes, uterus, and other affected tissues such as colon tissue. Adjuvant chemotherapy uses drugs to kill cancer cells that may have spread to other parts of the body [20,21]. These procedures and their limitations are a major burden for patients. Clinical trials and new therapies that explore innovative approaches, including targeted therapies and immunotherapy, are much needed to improve outcomes for women with this disease. Additionally, ongoing efforts to raise awareness about the symptoms and risk factors of ovarian cancer are crucial in promoting early detection and better overall survival rates.

### 1.3. HGSOC Tumor Microenvironment Presents Obstacles to Treatment

Current immunotherapeutic approaches have not been as promising as for other cancers due to the unique tumor microenvironment (TME) of ovarian cancer that facilitates efficient metastasis and dramatically impairs immune surveillance. Several comprehensive reviews discuss the composition and effects of the ascites TME on ovarian cancer progression and treatment [22,23,24,25,26,27].

HGSOC cells mainly metastasize within the peritoneal cavity [28]. The transition of epithelial ovarian cancer cells (EOCs) to a mesenchymal phenotype (EMT) involves aberrant expression of adhesion molecules [29,30] resulting in the loss of tight junctions and a more invasive behavior [31]. Cells that exfoliate from the primary tumor survive detachment by forming spheroids composed of tumor and non-tumor cells that can adhere to the mesothelium, omentum, and organs within the peritoneal cavity [32].

Peritoneal membrane permeability and angiogenesis, induced by EOC overexpression of vascular endothelial growth factor (VEGF), contributes to the accumulation of ascites fluid [33]. More than 90% of patients with stage III and IV ovarian cancer develop ascites fluid, the components of which constitute the TME [34]. The ascites TME contains diverse cell types including mesothelial, endothelial cells, immune cells, adipocytes, and fibroblasts. Interaction between tumor cells and the TME can occur through direct cell contact, soluble molecules, or exosome vesicles released by cells. The crosstalk among TME components shapes cellular phenotypes ultimately determining tumor progression or suppression.

#### 1.3.1. Anti-Tumor Immune Components in the TME

The migration of immune cells to the EOC tumor is orchestrated by various cytokines and chemokines (reviewed in [35,36,37]). Chemokine CCL5, constitutively expressed by EOC cells, induces the infiltration of CD8 T cells [38]. Interaction with peptides presented by MHC I on tumor cells induces CD8 T cells to produce cytolytic factors, granzyme and perforin, and cytokines IL-12, IL-2 and IFNγ that act to kill tumor cells. This immunogenic cell death along with genome instability [39] releases danger signals (DAMPS) [40] that attract antigen presenting (APC) and innate natural killer (NK) cells. In this environment, tumor-associated antigens (TAAs) can be presented to activate and amplify cytolytic T cell and B cell anti-tumor responses. Prevalent TAAs in HGSOC include NY-ESO-1 [41], MAGE [42], p53 mutation, and WT1 [43,44].

Interferons play an important role in the TME by regulating the gene expression of tumor infiltrating lymphocytes (TILs) [37]. Plasmacytoid dendritic cells, CD8 T cells, NK cells and T cell helper 1 (Th1) CD4 T cells are major sources of IFNγ. Anti-tumor M1 macrophages are induced by IFNγ and lipopolysaccharides [45]. IFNγ induces myeloid cell CXCL9 secretion, which cooperates with CCL5 to enhance lymphocyte recruitment [38]. IFN I induces CXCL13 expression in tumors, which correlates with the generation of tertiary lymphoid structures and infiltration of CD4, CD8 T cells and CD20 B cells [46].

Activated immune cells clearly have the potential to effect immune surveillance and eliminate or inhibit tumor growth. It is the relative resistance to re-programming by pro-tumor elements of the TME that determines the efficacy of the immune response.

#### 1.3.2. Pro-Tumor TME Components

The cellular components of the ascites TME include EOCs and spheroids, T cell subsets, tumor-associated macrophage (TAM), cancer-associated fibroblasts (CAFs), and myeloid-derived suppressor cells (MDSCs). Ascitic fluid facilitates the spread of tumor cells within the peritoneum to form metastatic lesions.

Interactions among cells, leading to altered phenotypes of tumor and immune cells, are mediated by cytokines, chemokines, and other factors, which bind to receptors that activate or inhibit signaling pathways. EOC cells release exosomes containing proteins, miRNA, or cytokines that can further activate tumor cells or reprogram fibroblasts or immune cells [47]. Macrophages can be polarized to an M2 pro-tumor phenotype [48] and CAFs can secrete cytokines that induce the metastasis and EMT of cancer cells [49].

The malignant ascites TME is hypoxic and has low levels of glucose and other nutrients [50,51,52,53]. Tumor hypoxia induces chemokines to recruit myeloid-derived suppressor cells (MDSCs), regulatory T cells (Tregs) and TAMs that create an immunosuppressive environment [51]. The TME impedes T cell function by inhibiting T-cell mitochondrial biogenesis and decreasing the production of bioenergetic intermediates [52,53]. Compared with peripheral T cells, higher levels of inhibitory co-receptors, such as LAG-3, PD-1, TIM-3, and CTLA-4, are found on T cells within the TME [54].

Major cytokines and chemokines and signaling factors that affect tumor progression and immune cell functions are presented in Table 1 and Table 2. The tumor immune microenvironment of ovarian cancer is considered “cold” compared with “hot” cancers that are characterized by a relatively high preexisting immune cell infiltration such as occurs with melanoma or lung cancers that respond successfully to immunotherapy. The ultimate immunotherapy success to cure many cold cancers, including ovarian cancers, depends on the relative ability to turn cold TME into hot [55,56].

### 1.4. Oncolytic Viruses as a Potential Therapeutic Strategy

Oncolytic viruses represent an evolving field of research for the treatment of ovarian cancer due to their ability to target and directly kill cancer cells while synergistically stimulating the body’s own anti-tumor immune response that also protects from tumor recurrence. Oncolytic viruses can also carry therapeutic genes to enhance their anti-tumor effects. These vectors are called “armed” oncolytic viruses. The expression of these genes is fully dependent on the replication of the virus [93,94]. Unlike gene therapy and gene editing, which needs to be customized for each patient, oncolytic viruses offer a targeted, yet multifaceted approach that could potentially improve outcomes for patients with this challenging disease.

Different types of oncolytic viruses have been investigated in preclinical and clinical studies for ovarian cancer treatment [95]. These viral vectors can be modified to target and kill ovarian cancer cells while stimulating immune responses. Table 3 lists studies of recent oncolytic virus-mediated therapies. The genomes of oncolytic viruses can consist of double-stranded DNA, single-stranded, negative sense RNA or single-stranded, positive sense RNA. Table 4 shows current clinical trials.

## 2. Sindbis Virus as a Novel Approach to Ovarian Cancer Treatment

### 2.1. Sindbis Is a Prototypic Alphavirus

Sindbis virus (SV), an enveloped, single-stranded, positive sense RNA virus, is a member of the alphavirus genus, *Togavirus* family [116]. In depth reviews of alphavirus structure, expression, infection, replication, and evolution have been published [116,117,118].

Alphaviruses have many attributes that render them advantageous for gene expression vectors: (1) they exhibit a broad host range for mammalian cell infection [119]; (2) RNA genomes, which mimic mRNA, quickly form replication complexes in the cytoplasm of infected cells; (3) the lack of DNA intermediates avoids the risk of insertional chromosome mutagenesis [120,121,122]; (4) approximately 10^6^ copies of viral RNA, coupled with a strong subgenomic promoter, enable very high expression levels of recombinant protein [120,123]; and (5) vectors are easy to manipulate and can accommodate at least 8000 bp of heterologous mRNA [124].

### 2.2. Alphavirus Vectors as an Oncoviral-Mediated Therapy

The properties of alphaviruses that make them suitable for cancer therapy include: (1) cytopathicity causing infected cells to die by apoptosis [125,126,127]; (2) tumor-associated antigens (TAAs) released from apoptotic bodies induce immune responses via cross-priming [128,129,130]; (3) double-stranded RNA replication intermediates elicit “danger signals”, activating antiviral pathways that stimulate innate immune responses and enhance adaptive immunity [131,132,133,134,135,136,137]; (4) alphaviruses infect lymph nodes [129,138,139] and dendritic cells [140,141]; (5) transmitted via insect bites, alphaviruses are blood-borne allowing recombinant vectors to be systemically delivered in vivo [116]; and (6) repeated administration of vectors remains efficacious [142,143,144].

### 2.3. Vector Safety

**Figure 1:**
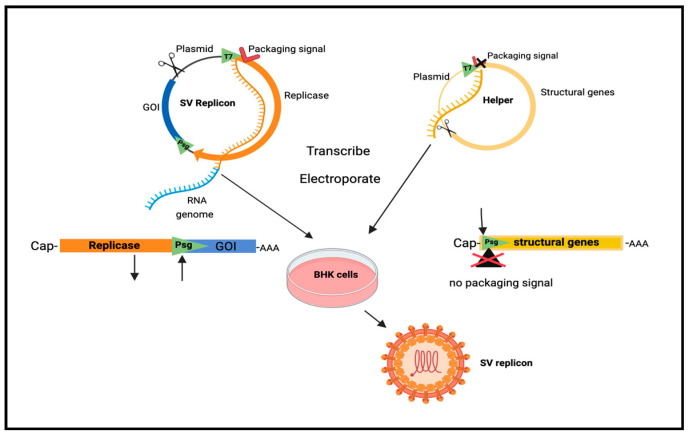
Preparation of SV vector. Plasmids are linearized, transcribed by T7 polymerase and capped in vitro. Transcripts are electroporated into BHK cells and viral vectors harvested from media [148]. T7, transcription promoter; Psg, Sindbis subgenomic promoter; GOI, gene of interest; AAA poly A tail; BHK, baby hamster kidney cells. Created with Biorender.

In nature, SV has the safest profile among alphaviruses with mostly asymptomatic infections causing mild fever, rash or arthralgia [145,146,147]. In addition, for added safety SV-derived vectors can be produced in a manner that prevents the amplification of virus particles (Figure 1). The cDNA sequence of the RNA genome is split into two segments and cloned into separate plasmids. A replicon DNA plasmid encodes the non-structural replicase proteins and contains a strong subgenomic promoter to express heterologous “genes of interest”. A helper DNA plasmid, encoding the viral capsid and envelope proteins, lacks a packaging signal so that vector particles only contain the replicase and heterologous genes and can undergo only one round of infection [120,123,148].

### 2.4. Sindbis Virus Selectively Targets and Replicates within Ovarian Cancer Cells While Sparing Healthy Cells

**Figure 2:**
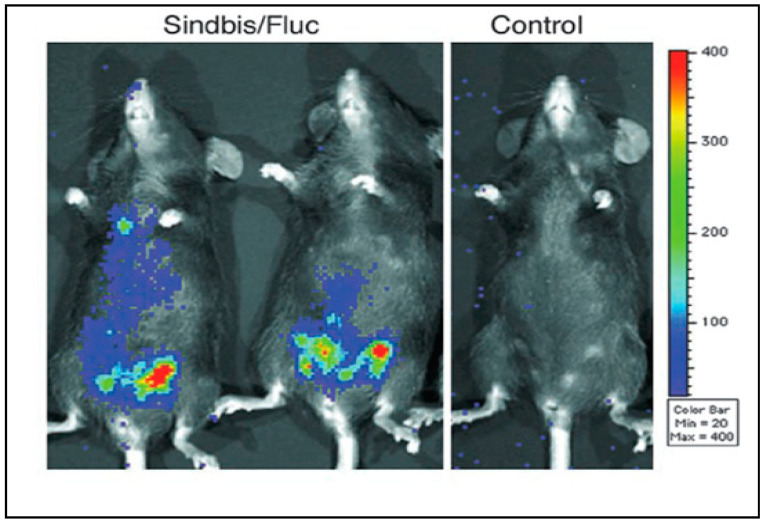
SV/Fluc vectors detect syngeneic MOSEC metastases in the peritoneum of immunocompetent C57BL/6 mice. Left panel: Four weeks after intraperitoneal injection (i.p.) of 1 × 10^7^ MOSEC cells, mice were treated with a single i.p. injection of SV/Fluc vectors (~10^7^ transducing units) and IVIS imaged the next day. Tumor free control mice were injected with SV/Fluc and imaged in parallel (Right panel) [125].

Immunohistochemical and bioluminescent in vivo imaging studies using SV vectors that express reporter genes indicate the specific targeting of tumor and metastatic cells in both murine xenotropic and syngeneic ovarian cancer models [125,149]. Figure 2 shows that SV vector expressing Firefly luciferase (SV/Fluc) localizes to tumors and metastases in a syngeneic C57BL/6 MOSEC ovarian cancer model [150] while non-tumor bearing control mice show no significant bioluminescent signals. Similar results were observed in a nude mice human ovarian cancer model systemically treated with replication-positive SV [115].

Sindbis virus has been shown to bind with the 67 KDa high affinity laminin receptor (LAMR) protein [151,152]. LAMR has a high affinity for laminin, a major component of cell basement membranes that plays an important role in cellular adhesion, morphology, differentiation, and migration (reviewed in [153]). The presence and conservation of LAMR among many distantly related species is consistent with the broad host range of the Sindbis virus. The overexpression of LAMR by cDNA transfection into Baby hamster kidney (BHK) cells increased the level of SV infection [152] while LAMR antisense RNA [152], silencing siRNA [125] or short hairpin shRNA [154] decreased infection.

Although the exact mechanism of SV infection via LAMR has not been elucidated (reviewed in [155]), it has been determined that the glutamic acid residue at amino acid position 70 of the envelope E2 protein is critical for in vivo tumor targeting [156]. The overexpression of the 67 KDa LAMR occurs on the surface of many human cancer cells [157,158,159,160,161,162] including those of ovarian origin [162,163]. LAMR monoclonal antibody binding studies suggest that tumor cells have varying degrees of unoccupied cell-surface LAMRs that conceivably result from the increased biosynthesis of LAMR or the dissolution of the surrounding basement membrane and extracellular matrix by tumor cells [164,165,166,167]. It is thus plausible that SV uses unoccupied LAMRs as fortuitous binding sites on tumor cells. Other SV receptors have been more recently discovered. The natural resistance-associated macrophage protein (NRAMP), a divalent metal transport protein, has been identified as an SV receptor in Drosophila and the vertebrate homolog, NRAMP2, as an SV receptor in mammalian cells [168]. VLDLR and ApoER2, members of the low-density lipoprotein family, were found to be receptors for certain alphaviruses including SV [169]. Affinity purification followed by mass spectrometry identified the CD147 membrane protein as a receptor for SV and other alphaviruses in human cells [170]. Notably, CD147, which has been found to be overexpressed on many cancer cells and cells within the tumor microenvironment, plays a role in tumor proliferation and the inhibition of apoptosis [171]. The protumor effects of CD147 may potentially be obviated by SV binding, infection, and cell lysis.

## 3. Mechanisms of Action against Ovarian Cancer Cells

SV vector cancer therapy can involve several processes. SV replication complexes produce abundant RNA molecules with double stranded RNA intermediates triggering “danger signals” [40]. The cytotoxic effects of SV infection result from the inhibition of cellular protein translation, activation of a stress response, and ultimately an apoptotic cascade [126]. The expression of tumor-associated antigens (TAAs) or immunomodulatory agents can augment anti-tumor effects.

### 3.1. Immunotherapeutic Effects of SV Vectors

The ability of SV vector treatment to stimulate a rapid influx of activated innate natural killer cells (NK) was demonstrated using a xenotropic ES-2 human ovarian cancer [172] model in SCID (severe combined immunodeficiency) mice, which lack T and B cells [133]. Significant inhibition of tumor growth and increased survival were observed. The incorporation of IL-12 into the SV vector enhanced the therapeutic effect by inducing IFNγ secretion from NK cells, which was shown to upregulate the expression of MHC class II on peritoneal macrophages promoting an M1 anti-tumor effect.

The potential of SV vectors to promote an adaptive immune response against tumors was studied in a syngeneic, immunocompetent BALB/c CT26 colon carcinoma tumor model [129]. As CT26 cells are not susceptible to SV infection, this model separated SV oncolytic activity from potential immunogenic effects. A bioluminescent signal was first observed in the mediastinal lymph nodes that drain the peritoneum 3 h after SV/Firefly luciferase i.p. injection. When CT26 TAAs were expressed by SV vectors, an influx of activated CD8 T cells to the peritoneum occurred within one week. Effector and memory CD8 T cells were generated correlating with long-term survival. The cytolytic activity of immune cells released endogenous CT26 TAAs that were shown to be engaged by CD8 T cell specific tetramers revealing that SV vectors can be therapeutic via epitope spreading without oncolytic tumor targeting.

### 3.2. Combination of IL-12 and OX40 Agonistic Antibody

Although many oncolytic virus vectors have been designed for the local delivery of IL-12 in preclinical trials, only oncolytic Herpes virus (OHSV-IL-12) has progressed clinically [173]. Improved therapeutic outcomes have required a synergistic or additive combined approach [174,175,176,177,178,179]. SV vectors expressing IL-12 cytokine have also been shown to have greater tumor-killing activity but have not been curative [133,180,181,182].

The observation that IL-12 increases the presence of OX40 (CD134) on the surface of CD4 T cells [181,183] prompted the study of a combined anti-tumor capacity. IL-12 activates T cells, stimulates the production of IFNγ and increases the expression of OX40 on effector CD4 T cells [183]. The combination of SV.IL-12 with an agonistic antibody to OX40 exhibits strong therapeutic efficacy in CT26, colon, and MyC-CaP, prostate carcinoma, models [181]. The transcriptome and metabolic reprogramming of T cells drove the development of activated effector T cells with enhanced tumor infiltration and anti-tumor capacity within the TME.

OX40 is a member of the tumor necrosis family that is expressed on activated T cells [184]. OX40 promotes the clonal expansion, differentiation, and survival of CD4 Th1 helper cells, which produce IFNγ and IL-2 cytokines [185,186,187,188,189] that sustain the survival of primed CD8 T cells [184,190]. The co-expression of OX40 with ICOS on follicular T helper cells (Tfh) facilitates the differentiation of antibody-producing B cells and long-lived plasma cells from germinal center B cells [191]. In addition, OX40 signaling represses regulatory T cells (Treg) by downregulating the expression of Foxp3 [192].

### 3.3. SV.IL12 and Agonistic Anti-OX40 as an Ovarian Cancer Therapy

The collaborative effects of SV.IL12 and anti-OX40 agonistic antibody (⍺OX40) were investigated using the C57BL/6 mouse MOSEC/ID8 model [150]. The MOSEC cells, which are susceptible to SV infection, were modified by cell passaging to achieve a more reproducible tumor engraftment and by transduction with firefly luciferase for in vivo imaging (MOSEC.Fluc.p11) [114]. MOSEC.Fluc.p11 cells (2.5 × 10^6^) were i.p. injected into C57BL/6 albino mice and within 7 days, tumors were observed. Treatment began on day 8 by i.p. injections of SV.IL-12 (10^7^ TU/mL, 0.5 mL), 4 times per week for 4 weeks and ⍺OX40 (250 µg/mouse), 3 times per week for 3 weeks. As observed for other cancer models, combined therapy increased survival [181], however, in the MOSEC model SV.IL-12 was almost as effective.

Treatment with SV.IL-12, ⍺OX40 and SV.IgGOX40 increased the migration of immune T cells into MOSEC.Fluc.p11 tumors within 7 days, as shown by the overlapping of multiplex immunofluorescence staining of CD8 and CD4 T cells with Ki67 and granzyme B markers for proliferating cytotoxic T cells [114]. Significantly more lysis of tumor tissue was observed when ⍺OX40 was delivered by SV.⍺OX40 vector presumably as SV infection of MOSEC cells produce high levels of and ⍺OX40 to stimulate a cytotoxic immune response and SV vector induced apoptosis.

The immune function of T cells require a high metabolic state. The ascites TME, characterized by hypoxia, acidosis, and low nutrient levels, impairs the metabolism and function of T cells (reviewed in [50,193,194,195]). The metabolic profiles of splenic T cells were measured by Agilent Seahorse technology. Only SV.IL-12 combined with ⍺OX40 provided T cells with a spare respiratory capacity and a higher basal energetic state [114].

**Figure 3:**
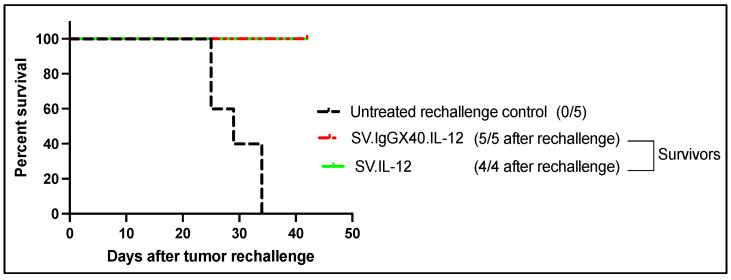
Mice treated with either SV.IL-12 or SV.IgGOX40.IL-12 at day 140 were rechallenged with MOSEC cells. The survivor mice were protected from recurrence after rechallenge [114,148].

To examine sustained protection from MOSEC growth, mice were treated with either SV.IL-12 or SV.IgGOX40.IL-12 and at 140 days, surviving mice were rechallenged with MOSEC cells (Figure 3). Naive control mice, inoculated with tumor cells at the same time as the rechallenged mice, succumbed after 35 days while both vectors provided long-term tumor suppression. The ability of SV.IL-12 to stimulate OX40 expression on T cells may account for the efficacy of both SV.IgGOX40.IL-12 and SV.IL-2 in this cancer model [114].

In vivo antibody depletion studies indicated that while both CD4 and CD8 T cells are important for SV.IgGOX40.IL-12 efficacy, CD4 T cells appear to play a more pivotal role [114]. This observation coincides with previous results showing that SV.IL-12 and ⍺OX40 elevate and sustain cytotoxic CD4 T cells [181]. Effector CD4 T cell have been increasingly recognized for anti-tumor activity, independent of their helper function [196,197].

Transcriptome analysis, performed for untreated MOSEC tumors vs. tumors treated with SV empty vector, SV.IL-12, SV.⍺OX40 or SV.IgGOX40.IL-12 showed distinct gene expression profiles for all treatment groups. Empty SV vector showed the lowest number of differentially expressed genes implying that the “armed” vectors were deployed at tumor sites. Pathway and network analysis showed the downregulation of genes involved with DNA replication, transcription, and cell division correlating with the elimination of tumor cells. Upregulated genes were predominantly related to immune response pathways most likely correlating with infiltrated lymphocytes.

**Figure 4:**
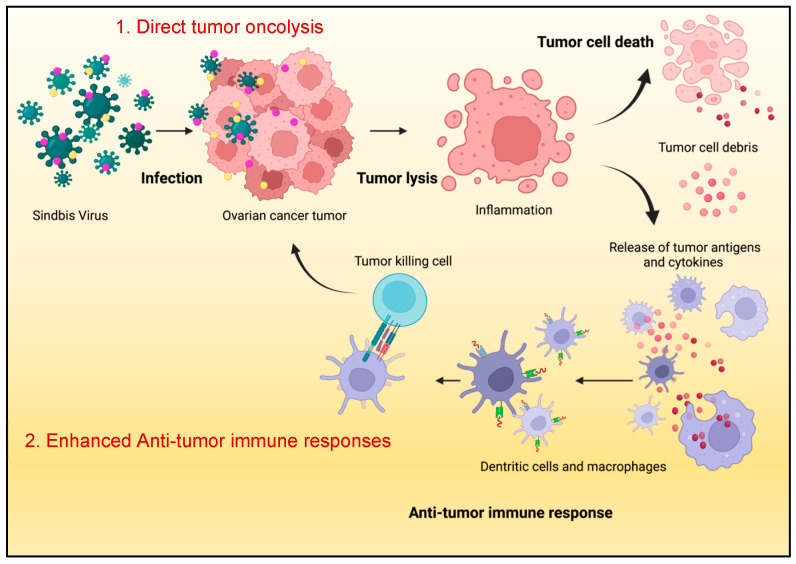
Summary of synergistic immune stimulating anti-tumor mechanism of armed SVs via (1) direct tumor oncolysis and (2) tumor influx of activated immune cells that enhance the anti-tumor response in the tumor microenvironment (TME) [114].

The modulation of anti-tumor immune responses is illustrated in Figure 4. The ability of SV.IgGOX40.IL-12 to systemically target metastatic tumors while altering the transcriptome signature and metabolic program of T cells, increasing their capacity to infiltrate the repressive TME, renders these vectors a promising therapy for ovarian and other types of cancers.

## 4. Summary

The unique TME of HGSOC complicates treatment strategies. More than 90% of stage III/IV patients develop malignant ascites fluid. Crosstalk among the myriad of the components of the ascites TME modulates the phenotypes of tumor and immune response cells. The goals of oncolytic virus-mediated therapies must include tumor targeting and killing, as well as, the skewing of the TME toward a strong immune response. Several recent studies and clinical trials have been presented.

A SV vector platform has been developed combining the expression of IL-12 and an agonistic antibody targeting the co-stimulatory OX40 receptor. The SV.IgGOX40.IL-12 vector decreases the tumor burden in ovarian cancer mouse models, increases survival and provides protection against tumor rechallenge. A notable influx of immune cells into the tumor microenvironment occurs. Treatment efficacy is associated with the transcriptional reprogramming of T cells leading to the expression of immune response genes and metabolic alterations that result in higher energy states.

The lack of clinical trials involving SV vectors, thus far, makes it difficult to evaluate their transition from animal models to human patients. Translation to clinical applications is feasible as SV vectors can be produced and purified under Good Manufacturing Practice (GMP) standards, ensuring high titers (10^11^ transducing units per milliliter (TU/mL)) to compensate for dilution in the bloodstream or TME. Vectors might be administered directly to patients or after the debulking of tumors and ascites where they may have a greater chance of preventing a suppressive TME. Treatment efficacy may require multiple doses. While alphaviruses are not highly immunogenic, the requirement for many doses could pose challenges and may lead to patient reluctance to undergo treatment. The ease of SV vector modifications, however, will allow continued optimization.

## 5. Future Perspectives

The lytic activity of oncolytic viruses, which selectively replicate in tumor cells, initiated their role as tools for cancer treatment. Ultimately, lytic effects were observed to reshape “cold” into “hot” tumors that are more responsive to immunotherapies. Table 3 and Table 4 present several families of oncolytic viruses that have been studied in combination with chemotherapy, checkpoint inhibitors, and other immunomodulatory agents for the treatment of ovarian cancer. Several oncolytic viruses have also been genetically modified to express therapeutic cargos.

Future studies should identify and optimize interactions between oncolytic viruses and the immune system within the TME. Epigenetic changes to cancer cells and effects on viral infection and replication should also be considered (reviewed in [198,199]). Epigenetic modifications of the genome, which include DNA methylation and histone acetylation, are often deregulated in tumors leading to cell proliferation. The inhibition of these modifiers have been shown to attenuate cellular anti-viral response, promote cancer cell cycle arrest and apoptosis and potentiate the immune response. The inhibition of histone deacetylase (HDACi) has been shown to augment adenovirus oncolysis in cisplatin-resistant ovarian cancer cells [200].

The considerable heterogeneity within patients with ovarian cancer involving genetic, epigenetic, and immunological systems presents a challenge to therapy. Oncolytic viruses combined with the modulators of the immune system, epigenome, and chemical drugs can provide powerful weapons in the arsenal against ovarian cancer.

## Figures and Tables

**Table 1:** Pro-tumor cytokines and chemokines.

Cytokine	Origin	Effects	Refs.
IL-1β	EOC	↑ tumor aggressiveness; T regs; ↓ NK and mT cells	[57,58]
IL-6	EOC, CAFs, TAMs, adipocytes	↓ DC maturation; immunosuppression; ↑ MDSCs activates cell signaling pathways	[59,60]
		that ↑ cell proliferation; ↑ EMT	[59,60,61]
IL-8	EOC, CAFs	↑ angiogenesis, ↑ cancer cell migration	[62,63]
IL-10	EOC, T regs, TAMs	↑ MDSCs; ↓ CD8 T cell function; ↑ M2 polarization	[64]
IL-13	EOC, CD4 Th2	↑ EOC invasion and metastasis	[65,66]
TGFβ	MDSCs, CAFs, TAMs	↑ EMT; activates JAK/STAT-3; ↑ MMPs; ↑ Tregs; ↑ metastasis; ↑ VEGF	[67,68,69,70]
CSF-1	EOC	recruit and activate M2 TAMs	[45,71]
TNF⍺	EOC	↑ metastasis; ↑ VEGF	[72]
**Chemokine**	**Origin**	**Effects**	**Refs.**
CCL1	EOC, CAFs, TAMs	drives MDSCs to tumor	[73]
CCL2	EOC	recruit and activate M2 TAMs	[71]
CCL18	EOC	↑ EOC invasion and metastasis	[74]
CCL20	EOC	↑ metastasis	[75]
CXCL12	EOC, stromal cells	↑ EOC invasion and metastasis	[76,77]

EOCs, epithelial ovarian cancer cells; Tregs, regulatory T cells; NK, natural killer cells; mT cells, memory T cells; CAFs, cancer associated fibroblasts: TAMs, tumor associated macrophage; DC, dendritic cell; MDSCs; myeloid derived suppressor cells; EMT, epithelial to mesenchymal transition; M2, pro-tumor macrophage; JAK/STAT-3, Janus kinases/signal transducer and activator of transcription; MMPs, matrix metalloproteinases; VEGF, vascular endothelial growth factor. ↑, increased; ↓, decreased.

**Table 2:** Pro-Tumor signaling factors and pathways.

Components	Origin	Effects	Refs.
VEGF	EOC, mesothelial cells	↑ peritoneal, vascular permeability; ↑ MMP	[10,15,78]
CA-125(MUC16)	EOC	EOC adhesion to mesothelial cells; ↑ invasiveness	[79,80]
IDO	MDSCs,	↑ T regs; ↓ NK function	[81,82]
COX2/PGE2	EOC	↑ MDSCs; ↓ TIL recruitment; ↑ Tregs	[83,84]
CD95L (Fas)	EOC exosomes	kills immune cells expressing CD95R	[85]
GD3 gangliocide	EOC	suppresses the innate immune response	[86]
**Pathways**	**Origin**	**Effects**	**Refs.**
NFkB	EOC, macrophage	↑ growth; ↑ invasiveness; ↑ angiogenesis; immunosuppression	[21,87]
STAT3	EOC, CAFs	key role in tumor progression ↑ angiogenesis ↓ apoptosis;	
		↑ M2 polarization ↑ EMT in spheroids ↑ T regs; ↑ MDSCs; ↑ proliferation and metastasis	[88,89,90]
PI3K/AKT	EOC, CAFs	↑ EMT, OC cell growth	[91]
FAK	EOC	↓ TILs	[92]

CA-125(MUC16), cancer antigen, mucin; IDO, indolamine 2,3-dioxygenase; COX-2, cyclooxygenase-2; PGE2, prostaglandin E2; TIL, tumor infiltrating lymphocyte; NFκB, nuclear factor κB; PIK, phosphinositide 3-kinase; AKT, protein kinase B; FAK, focal adhesion kinase. ↑, increased; ↓, decreased

**Table 3:** Studies of Oncolytic Virus-mediated Treatment of Ovarian Cancer.

Virus Type	Family	Designation	Virus Modification/Combined Treatment	Refs.
dsDNA	*Adenoviridae*	OAd-MUC16-BiTE	expresses bispecific Ab to MUC16 and CD3	[96]
		Ad5 ΔE1b, E3	combination with anti-PD-1, CSF-R1 inhibitor	[97]
		AR2011(h404)	expresses hCD40 and h41BBL combined with cisplatin	[98]
		M11	capsid contains tumor targeting peptide TMTP1 expresses truncated BID (mitochondrial apoptotic protein) combined with cisplatin	[99]
		Ad-VT	hTERT tumor specific promoter, expresses apoptin	[100]
		TILT-123	expresses hTNF ⍺ and hIL-2	[101]
		CRAd	MUC16 promoter transactivation region targets cells with high CA-125 expression	[102]
		TILT-321	expresses bispecific MUC1 and CD3	[103]
	*Herpesviridae*	OV-αCD47-G1	binds to Fc-receptors on NK and macrophage combined with PD-L1 antibody	[104]
		NG34ScFvPD-1	expresses single chain PD-1 antibody combined with PI3K inhibitor (LY294002)	[105]
	*Poxviridae*	OV-CXCR4-A (Vaccinia)	CXCR4 antagonist in the context of the Fc portion of murine IgG2a	[106]
		OrfV	monotherapy; stimulates NK cells	[107]
ss(−)RNA	*Rhabdovirus*	VSVΔM51	attenuated VSV combined with α-galactosylceramide-loaded DCs	[108]
		VSVΔM51	attenuated VSV combined with T-DM1 (Kadcyla^®^)	[109]
		MeV-Stealth	MeV pseudotyped with CDV envelope modified to target CD46	[110]
		oMRB	expresses OVA as a TAA	[111]
	*Paramyxovirus*	NDV(F3aa)-GFP	expresses thrombospondin peptide (3TSR) combined with MIS416Vax adjuvant for vascular normalization	[112]
ss(+)RNA	*Retroviridae*	oFV	infects slow dividing cancer cells latent in quiescent cells, replicates upon cell division	[113]
	*Alphaviridae*	SV.IgGOX40.IL-12	expresses IL-12 and agonistic antibody to OX40	[114]
		SIN AR339	replication competent Sindbis virus	[115]

VSV, vesticular stomatitis; MeV, measles virus; oMRB, Maraba virus; NDV, Newcastle disease virus; oFV foamy virus; SV Sindbis virus; dsDNA, double-stranded DNA; ss(−)RNA, single-stranded, negative sense RNA; ss(+)RNA, single-stranded, positive sense RNA.

**Table 4:** Clinical trials of oncolytic virus mediated treatment of ovarian cancer.

Name	Type	NCT ID	Phase	Investigations
MV-CEA or NIS	measles	NCT00408590	I	safety and toxicity of MeV expressing different TAAs
MV-NIS	measles	NCT02068794	I/II	i.p. administration for infection of mesenchymal stem cells
MV-NIS	measles	NCT02364713	II	i.p. administration combined with chemotherapy of choice
TILT-123	adenovirus	NCT05271318	I	encodes TNF⍺ and IL-2 combined with Pembrolizumab
R130	herpes	NCT05801783	I	safety/efficacy of recombinant HSV I for relapsed/refractory OC
GL-ONC	vaccinia	NCT02759588	I/II	i.p. infusion +/− chemotherapy
Olvi-Vec	vaccinia	NCT05281471	III	safety and efficacy of adding Olvi-Vec to platinum and bevacizumab treatment

## Data Availability

All sequencing data that support the findings of this study will be. deposited in the National Center for Biotechnology Information Gene Expression Omnibus (GEO) and are accessible through the GEO Series accession number that will be provided and including all other relevant data included in the article, and further inquiries can be directed to the corresponding authors.
